# Magnetic Resonance Imaging to Detect Early Molecular and Cellular Changes in Alzheimer's Disease

**DOI:** 10.3389/fnagi.2016.00139

**Published:** 2016-06-16

**Authors:** Michael J. Knight, Bryony McCann, Risto A. Kauppinen, Elizabeth J. Coulthard

**Affiliations:** ^1^School of Experimental Psychology, University of BristolBristol, UK; ^2^Clinical Research and Imaging Centre, University of BristolBristol, UK; ^3^Research into Memory the Brain and Dementia Group, Institute of Clinical Neuroscience, University of BristolBristol, UK; ^4^North Bristol NHS TrustBristol, UK

**Keywords:** Alzheimer's, magnetic resonance imaging, shape analysis, relaxometry, diffusion tensor imaging

## Abstract

Recent pharmaceutical trials have demonstrated that slowing or reversing pathology in Alzheimer's disease is likely to be possible only in the earliest stages of disease, perhaps even before significant symptoms develop. Pathology in Alzheimer's disease accumulates for well over a decade before symptoms are detected giving a large potential window of opportunity for intervention. It is therefore important that imaging techniques detect subtle changes in brain tissue before significant macroscopic brain atrophy. Current diagnostic techniques often do not permit early diagnosis or are too expensive for routine clinical use. Magnetic Resonance Imaging (MRI) is the most versatile, affordable, and powerful imaging modality currently available, being able to deliver detailed analyses of anatomy, tissue volumes, and tissue state. In this mini-review, we consider how MRI might detect patients at risk of future dementia in the early stages of pathological change when symptoms are mild. We consider the contributions made by the various modalities of MRI (structural, diffusion, perfusion, relaxometry) in identifying not just atrophy (a late-stage AD symptom) but more subtle changes reflective of early dementia pathology. The sensitivity of MRI not just to gross anatomy but to the underlying “health” at the cellular (and even molecular) scales, makes it very well suited to this task.

## Introduction

Alzheimer's disease (AD) is the most prevalent form of dementia (Jorm and Jolley, 1998), with Mild Cognitive Impairment (MCI) often indicating a pre-AD phase (Albert et al., 2011), but even at this point, brain pathology has been accumulating for many years (Jack et al., 2013). Hallmark pathological changes in Alzheimer's disease (AD) are amyloid plaques and neurofibrillary tangles containing hyperphosphorylated tau (Perl, 2010) with associated cerebral amyloid angiopathy in over 80% of cases (Ellis et al., 1996).

Early diagnosis of dementia is becoming critical as emerging treatments may delay progression of disease only in the earliest stages (Liu-Seifert et al., 2015), *before* most patients are diagnosed. The annualized conversion rate from MCI to AD is around 10% (Ward et al., 2013), but many patients with symptoms of MCI will not develop dementia (even when there is some biomarker evidence of AD, Jack et al., 2016) and some will develop non-AD dementia. So symptoms alone cannot accurately predict those who have early stage AD. Current routine clinical tests include:

1. **Neuropsychology** reveals a pattern of deficits consistent with AD in the early stages, but changes are non-specific and an individual's baseline can be hard to ascertain;
2. **Structural imaging** Computed Tomography or MRI can identify groups with MCI likely to progress to AD (Risacher et al., 2009), but current implementations do not classify an individual with sufficient accuracy. Although generally well tolerated, MRI can be limited by symptoms of claustrophobia and presence of metal implants.
3. **Positron Emission Tomography (PET) and Single Photon Emission Computed Tomography** (O'Brien et al., 2014; Archer et al., 2015; Weiner et al., 2015) Amyloid PET can differentiate AD from healthy controls with over 95% accuracy (albeit with substantial false positive rate) and also identifies most people with MCI likely to develop AD (Vandenberghe et al., 2013). Although not in routine clinical use, there are also PET ligands that bind to phosphorylated-tau (FDDNP molecule (Small et al., 2006) and ^18^F AV1451) that have recently been shown to track progressive tau accumulation in aging and Alzheimer's disease Schöll et al. The expense of these tests is prohibitive in most centers.
4. **Lumbar puncture** Low amyloid and raised tau in cerebrospinal fluid (CSF) predict AD with 95% sensitivity and 87% specificity after 4–6 years' follow up (Hansson et al., 2006), but lumbar puncture is an invasive test.

MRI is widely available and patients are routinely imaged in diagnostic work up. Therefore, we predict that updated application of MRI will become increasingly important for accurate dementia diagnosis.

## Fundamental principles of MRI

MRI exploits magnetic properties of atomic nuclei with the quantum mechanical property of spin angular momentum, commonly known as “the spin.” Hydrogen (^1^H) is an abundant atom *in vivo* with a spin. As water molecules have two ^1^Hs and our body is ~70% water, ^1^H is an excellent reporter for MRI (sodium = ^23^Na is the other inherent nucleus used for MRI of living objects, not covered here). Thus, MR image detects water *in vivo* in a spatially-specific manner where MR signal is weighted to a given biophysical property of ^1^H according to the data acquisition procedure.

Clinical MRI of human brain uses scanners with magnetic field strength typically ranging from 1 Tesla (T) to 3T; in research settings magnets up to 9.4T (ultra-high field systems operate ≥ 7T) are used for humans. The strong magnet produces “net magnetisation” of ^1^H nuclei that results from “spin excess” in alignment in the magnetic field. The amount of net magnetisation scales up with the magnetic field strength so that the signal-to-noise ratio (SNR) in MR images increases roughly linearly with the field. So, there is a clear imperative toward higher magnetic fields as higher SNR can yield higher spatial resolution, improved temporal resolution in functional imaging, or improved access to microstructural information in diffusion imaging (at a constant spatial resolution).

Signal intensity in the MR image is chosen according to dynamic properties of the ^1^H nuclei, such as rotational and translational motions, that are weighted according to the MR data acquisition parameters. Rotational motion of nuclei is probed through MR relaxation, which is described by two time constants: the longitudinal relaxation time or T_1_ and the transverse relaxation time or T_2_. T_1_ and T_2_ are commonly exploited for structural MRI at macroscopic scale, also known as volumetry. It should be stressed that both quantitative T_1_ (Barazany and Assaf, 2012) and T_2_ (MacKay et al., 1994) can also provide data indirectly from tissue microstructure.

### Morphometry

In this section we will cover volumetry (which we define as volume analysis of macroscopic brain structures), cortical thickness and shape analyses [Voxel Based Morphometry or parcellation not reviewed (See Whitwell et al., 2012 for information; Yang et al., 2012)].

The volume of a tissue or subcortical structure in the brain is reflective of the number of neurons, dendritic processes or synapses (Schuff et al., 2009; Jack, 2012). Quantitation of volumes is therefore sensitive to various diseases and their progression. MRI is an excellent modality to quantify brain and subcortical volumes and is widely used in assessment of suspected MCI or AD (Jack et al., 2012; Weiner et al., 2015). MR volumetry generally uses 3D T_1_-weighted data sets, which can be acquired at a 1 mm isotropic resolution in around 5 min. Volumetric analysis based on such data can readily be automated, for which various fast and robust software packages exist (Dale et al., 1999; Tzourio-Mazoyer et al., 2002; Fischl et al., 2004; Jack, 2012) and this would enhance clinical practice if implemented in the near future.

Cortical thickness estimates (Li et al., 2015; Redolfi et al., 2015) are straightforward from 3D T_1_-weighted images. It should be noted that what is said about 3D T_1_-weighted images apply to scans acquired at clinical field strengths. At ultra-high fields T_1_ becomes longer which has to be accounted for in setting data acquisition parameters. The same brain region of two individuals may atrophy by the same amount, but localized to different particular subregions, yielding the same volume (and volume loss) but different symptoms for the afflicted individuals; shape analysis seeks to address this.

### Functional MRI

Functional MRI (fMRI) most commonly probes haemodynamic response, resulting from local decline in the ratio of deoxyhaemoglobin-to-oxyhaemoglobin and increase in blood volume elicited in response to “brain activation.” The so-called Blood Oxygenation Level Dependent (BOLD) signal localizes haemodynamic response to brain activation, the BOLD effect influencing the transverse relaxation rates T_2_ and ${\text{T}}_{2}^{*}$. Reduction of local magnetic gradients and increased blood volume prolong parenchymal T_2_(^*^), which is recorded as a positive BOLD signal by means of T_2_(^*^)-weighted MRI.

Haemodynamic responses due to experimental external events result in task-activated BOLD fMRI from which inferences have been made with regard to cognitive function. Fluctuations in BOLD without an external trigger, the so-called resting state fMRI, allows indirect inference about intrinsic brain connectivity and this may change as a result of neurodegenerative disease and is discussed below.

### Diffusion tensor imaging

**Figure 1:**
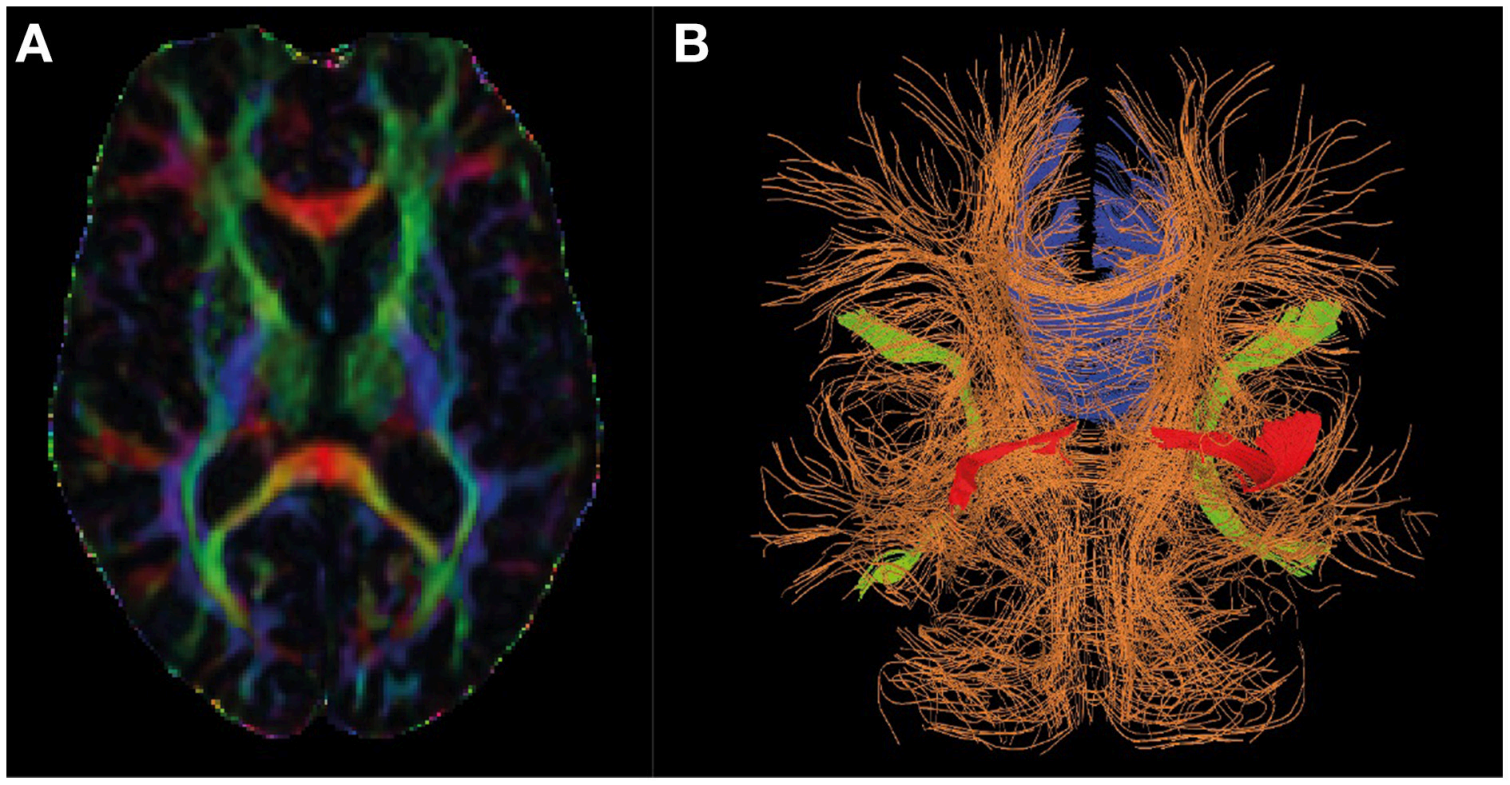
**Diffusion tensor MRI in Alzheimer's research**. Panel **(A)** shows the directional information encoded by diffusion tensor imaging by color (red: left-right, green: anterior-posterior, blue: head-foot) and fractional anisotropy (high intensity implies high fractional anisotropy). Panel **(B)** shows how the directional information may be represented in a deterministic tractography analysis, and shows the regions consistently identified and changing in AD (Blue: Corpus callosum, red: uncinate fasciculus, green: superior lateral fasciculus).

Diffusion tensor imaging (DTI) seeks to image the translational (thermal) motion of water, which is a tensor field (different in different directions and different at every voxel of the image) due to microstructural barriers to diffusion (e.g., cells/subcellular structures) constraining the motion of water differently along different directions (Basser and Jones, 2002). The translational motion of water is probed by sensitizing the MR signal to “diffusion” by means of magnetic field gradient-based approaches (see Figure 1 for an example). Because of this DTI can inform about the tissue microstructure at subvoxel resolution—it is the cellular and subcellular architecture which constrains the motion of water *in vivo*. Whilst DTI contains a wealth of information from water diffusion *in vivo*, analysis often focuses on the extraction of the mean diffusivity (MD) and fractional anisotropy (FA), referred to as DTI metrics below, as well as axial and radial diffusivity (Stebbins and Murphy, 2009; Amlien and Fjell, 2014; Zhang et al., 2014). The MD refers to the average rate of diffusion along the three axes of the diffusion tensor and is sensitive to water content and overall tortuosity (level of “constraint” of diffusion) of the environment. FA parameterises the extent to which diffusion is different along the three axes of the diffusion tensor, taking extreme values of zero when diffusion is the same in all directions (as in CSF) and unity when constrained entirely along a single direction (such as along a fiber tract). DTI can be used to estimate axonal density and diameter in white matter as indices of white matter integrity (Madden et al., 2012).

### MR spectroscopy

Magnetic resonance spectroscopy (MRS) seeks to observe a spectrum of resonance lines, each deriving from a unique spin state transition frequency. Although, there is much overlap of lines in the magnetic resonance spectrum of an *in vivo* system, which contains many molecules each with many spins, there are still enough well resolved resonances deriving from unique metabolites for it to be a very powerful method. A unique feature of ^1^H MRS is that it reveals cell-specific metabolite biomarkers, such as N-acetyl asparate (NAA) and myo-inositol (Amlien and Fjell, 2014). The intensities or integrals of spectral lines are directly informative of metabolite concentrations. MRS can be performed with spatial specificity, recording a spectrum from a voxel of choice within the brain (Oz et al., 2014).

### Relaxometry

Magnetic resonance relaxometry is the imaging of relaxation parameters such as T_1_, T_2_, ${\text{T}}_{2}^{*}$, and T_1ρ_, describing various aspects of the re-equilibration of the spin system after perturbation by radiofrequency pulses. Like diffusion parameters, they essentially describe the status of a tissue. Typically they take higher values when a tissue is “unhealthy” due to oedema (more motile water present) or when cells have been broken down, reducing dephasing of magnetisation, thus prolonging signal lifetimes quantified by T_2_ and ${\text{T}}_{2}^{*}$, and making water by and large more motile, which increases all relaxation parameters.

### Brain perfusion

Brain perfusion, which is driven by cerebral blood flow, can be detected by arterial spin labeling (ASL) MRI. In ASL MRI the arterial blood entering the brain is magnetically labeled either by radio frequency (RF)-field or RF-inversion pulse. The signal decline between the non-labeled (“control”) and labeled ASL images is proportional to the tissue perfusion and the signal difference can be converted with appropriate procedures into absolute units of blood perfusing a unit volume of tissue per minute (Alsop et al., 2015).

## Brain atrophy in AD and MCI

A slow decrease in total brain volume is part of physiological aging (Peters, 2006). Both white matter (WM) and gray matter (GM) decline in volume during normal aging (Sullivan et al., 1995; Salat et al., 1999; Peters, 2006; Scahill and Fox, 2007) with gray matter atrophy undergoing global atrophy accelerated in insula, superior parietal gyri, central sulci, and cingulate sulci and relative sparing in amygdala, hippocampi, and entorhinal cortex. In contrast, global white matter does not decline, but there are islands of accelerated decline particularly of white matter areas to frontal lobes and thalamus (Good et al., 2001). An increased rate of atrophy is predictive of AD (Jack, 2012), though also of other forms of dementia (Chan et al., 2001). An abnormal ratio of GM to cerebrospinal fluid (CSF) is also suggestive of a disease processes (Sullivan et al., 1995; Salat et al., 1999).

### Hippocampal atrophy in AD and MCI

Atrophy rates are different for different regions and tissues. In particular, the hippocampus often suffers particularly severe atrophy in AD (Jack, 2012), and often (though not always) before the outward display of cognitive symptoms. Volumetric analysis of the whole HC has been shown to be an effective biomarker for demonstrating short-term clinical decline in AD (Landau et al., 2010; Ewers et al., 2012; Lehmann et al., 2013; Jack et al., 2015) and also shows relative success at predicting conversion from MCI to AD (Schuff et al., 2012; Liu et al., 2013). Rate of atrophy has consistently performed well in comparison to other biomarkers and cognitive indices (Holland et al., 2012; Leung et al., 2013; Jack et al., 2015).

**Figure 2:**
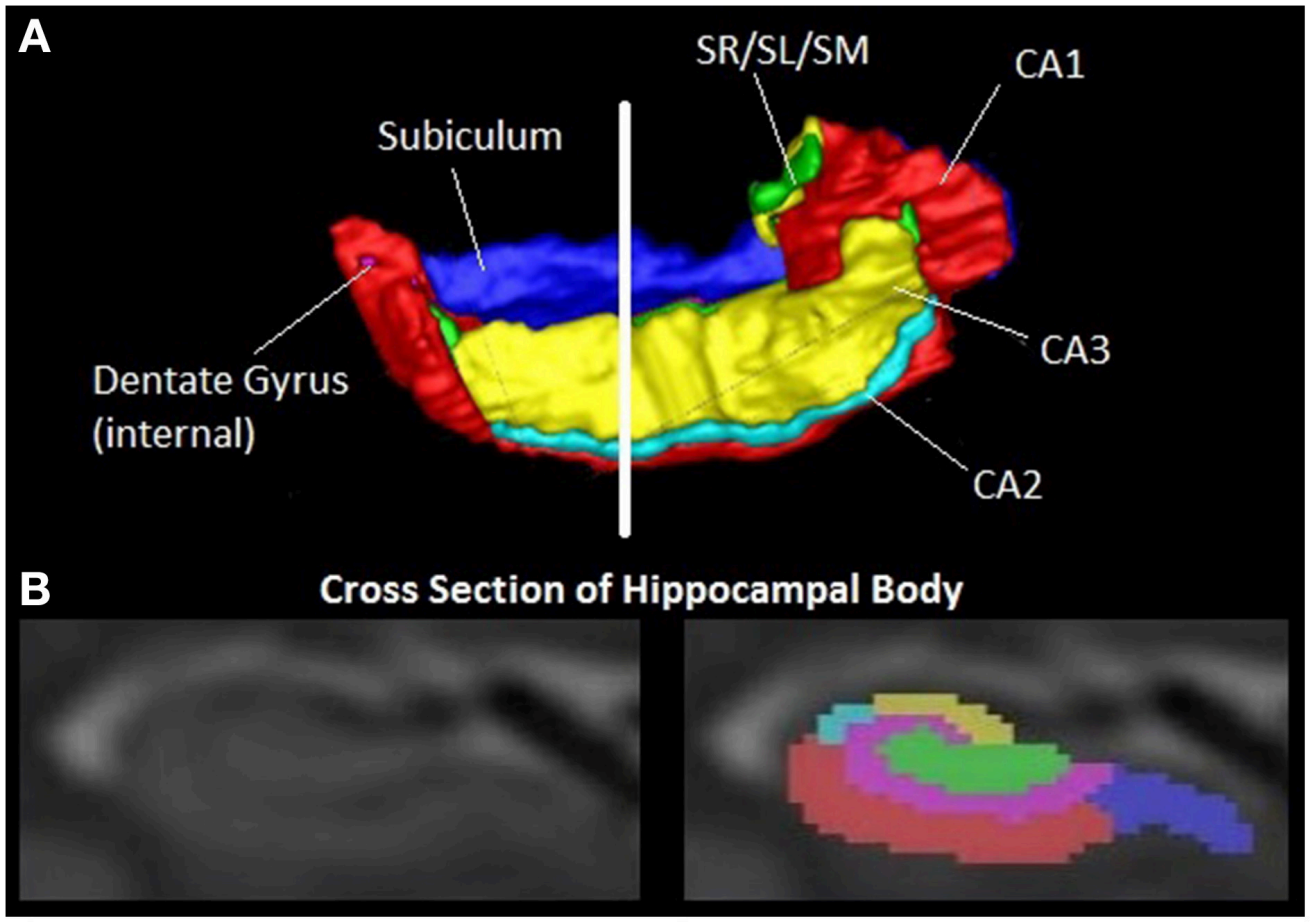
**The human hippocampus**. Image shows a 3D representation of the right hippocampus with six subfields and a coronal MR image of the hippocampal body with corresponding subfields. Segmentation as described in (Wood et al., 2015). Red = CA1, Light blue = CA2, yellow = CA3, blue = subiculum, pink = dentate gyrus, green = Stratum radiatum/stratum lacunosum/ stratum moleculare (SR/SL/SM).

However, the HC is a complex structure, composed of several subfields (Figure 2), distinct in terms of cellular content and connections with other brain regions (Duvernoy, 2005). Autopsy data show that these are differentially vulnerable to AD pathology (Braak and Braak, 1991; Jack et al., 2002; Silbert et al., 2003; Fearing et al., 2007; Zhang et al., 2015).

An increasing body of recent research indicates that the quantitation of individual subfield volumes has superior sensitivity and specificity in detecting and distinguishing AD and MCI, as well as other dementias (La Joie et al., 2013; Li et al., 2013; Maruszak and Thuret, 2014; Perrotin et al., 2015). An emerging picture is that the CA1 and subiculum subfields have the highest diagnostic power in detecting early AD (de Flores et al., 2015).

There is ongoing research to automate the labeling of hippocampal subfields in MR images (Yushkevich et al., 2015).

### Beyond the hippocampus

Beyond the HC, temporal and parietal volumes can identify healthy individuals who are at risk of future memory decline (Chiang et al., 2011). Other areas including subfields within the thalamus—a connectivity hub for the neocortex—are abnormal in established AD (Braak and Braak, 1991). A fascinating area to consider for the future is how brain network disruption including distributed brain areas might actually precede and even propagate AD pathology (Kapogiannis and Mattson, 2011; Sotiropoulos et al., 2013). Thus, metabolic and network biomarkers such as fMRI and DTI may become important. However, currently medial temporal and parietal regions have been shown to be individual regions most susceptible to changes of early AD (Heckemann et al., 2011).

The medial temporal area Entorhinal cortex (EC), in particular, is recognized as a region severely affected by AD pathology and is reported to be the most heavily damaged cortex in AD (Van Hoesen et al., 1991). EC atrophy is predicted to occur prior to hippocampal damage and is one of the earliest signs of disease manifestation. The rate of atrophy in the EC correlates with severity of cognitive symptoms (Li et al., 2012) and is considered predictive of conversion from MCI to AD (Devanand et al., 2007).

Furthermore, AD patients display cortical thinning in bilateral, frontal, parietal, and occipital lobes compared to controls and thinner cortex in parts of the bilateral parietal and precuneus region compared to frontotemporal dementia (Du et al., 2007). A negative correlation between cognitive performance and cortical thickness was also reported in these regions (Du et al., 2007), indicating that cortical thickness in these regions is a marker of disease severity.

## Beyond volume: Analysis of shape

The patterns of loss of cortical thickness from MRI agree with those seen in autopsies and appear in early phase of AD in the inferior temporal lobe cortex (Marshall et al., 2014), followed by peri-ventricular structures prior to a more widespread cortical thinning. Salat et al. reported that cortical thinning in AD is associated with decrease in contrast-to-noise-ratio (CNR) between gray and white matter in T_1_ images (Salat et al., 2011). The decrease in CNR is thought to result from microstructural changes in brain tissue, which are likely to precede loss of tissue volume and could be earlier indication of imminent pathology (Westlye et al., 2009; Callaghan et al., 2014).

Both local displacements and global shape alterations in the hippocampus have been shown to distinguish AD from MCI (Wang et al., 2003; Thompson et al., 2004; Ceyhan et al., 2011), and identify MCI to AD conversion rates with 80% accuracy (Costafreda et al., 2011). Involvement of the CA1 subfield has been consistently reported in the latter studies, particularly in the right hemisphere in agreement with detailed volumetric analysis and autopsy findings.

## Increased diffusivity and decreased fractional anisotropy are the hallmarks of MCI and AD

The general picture from the use of MD and FA as diffusion metrics is that MD is increased whereas FA is decreased in AD and MCI patients relative to controls. In a meta-analysis of AD and MCI studies conducted up to 2010 (Sexton et al., 2011), the largest MD effects were found to be localized to the HC, temporal and parietal regions. The largest FA decreases relative to controls have generally been found in the uncinate fasciculus, superior lateral fasciculus and corpus callosum. More recent work (Palesi et al., 2012; Hong et al., 2013; Jacobs et al., 2013) has continued to reinforce these findings. It has also been reported in recent studies that axial and radial diffusivity show widespread increases in both MCI and AD, and that these measures are more sensitive to dementia and cognitive impairment than FA (Shu et al., 2011; Nir et al., 2013). DTI has been shown to have superior patient/non-patient classification capability relative to volumetric analysis alone (Santillo et al., 2013) and in combination therewith (McMillan et al., 2014).

## DTI metrics and cerebrospinal fluid (CSF) biomarkers in AD and MCI

There has been work recently to relate levels of known AD CSF biomarkers Tau and Amyloid. Although a consensus has yet to be reached, there is an emerging picture that DTI is a powerful complement to the use of CSF biomarkers (Clerx et al., 2012).

CSF biomarkers have been variously related to increased hippocampal MD in AD (Bendlin et al., 2012; Douaud et al., 2013) and white matter FA in MCI (Selnes et al., 2013), with the general conclusion that DTI measures are superior predictors of AD than CSF biomarkers, and the use of DTI and CSF biomarker quantitation in concert is superior again.

## Relaxometry

A recent study quantifying T_1ρ_ in the medial temporal lobe (MTL) showed increased T_1ρ_ in MTL WM and GM in AD (Haris et al., 2011) and that its measurement, like DTI, surpassed the sensitivity of CSF biomarkers Tau and Aβ42 in diagnosing AD, though CSF biomarkers showed better specificity (Haris et al., 2015).

## Functional connectivity (or resting-state MRI)

There has been increasing interest in recent years in resting-state functional connectivity, where MRI is monitored by BOLD contrast. A large literature now exists on resting state fMRI applications in dementia (Zhou and Seeley, 2014). Whilst the results have not yet fully converged, recent reviews of applications in AD (Sheline and Raichle, 2013; Krajcovicova et al., 2014) note consistent observations of alterations to the default mode network, though many brain networks are variously implicated. Interestingly, the relationship between functional connectivity, brain volume and cognitive ability differs during lifespan with, for example, cortico-striatal functional connectivity being related to change in recall of episodic memory in older adults more than younger people (Fjell et al., 2015).

## Perfusion imaging and amyloid related vascular changes

An emerging picture in AD research is the role of cerebral perfusion, with vascular changes occurring early in disease progression (Mazza et al., 2011). In particular, global and regional cerebral hypoperfusion are associated with the disease, though debate continues as to whether this is a cause or effect (de la Torre, 2000). Either way, the observation that AD is thereby linked to modifiable vascular risk factors has attracted considerable attention. Hypoperfusion as measured by ASL has been consistently observed in the posterior cingulate, precuneus, inferior parietal, and lateral prefontal cortices (Alsop et al., 2010; Wolk and Detre, 2012; Wierenga et al., 2014). ASL findings compare well to FDG-PET (Musiek et al., 2012), with recent data also suggesting the capacity of ASL to add to diagnostic power, with distinct patterns of perfusion in different dementias (Binnewijzend et al., 2014).

As well as atherosclerotic changes in people with AD, blood vessels can be affected by cerebral amyloid angiopathy or damage from amyloid-modifying therapies (known as Amyloid Related Imaging Abnormalities–ARIA). Such changes include vasogenic oedema (visualized on T_2_ weight Fluid Attenuation Inversion Recovery Sequences) and microhaemorrhages (visible on ${\text{T}}_{2}^{*}$ sequences such as Gradient Echo) (Sperling et al., 2011).

## Spectroscopy

In the large literature on the use of ^1^H MRS in dementia, consistent findings in AD include significantly reduced NAA, the neuron-specific MRS biomarker, in the posterior cingulate and bilateral hippocampus (Murray et al., 2014; Wang et al., 2015). Similar to NAA, glutamate has been shown to be decreased in these brain structures (Alsop et al., 2010). The glial metabolite, myo-inositol, is increased in the posterior cingulate (PC) and parietal GM (Wang et al., 2015; Murray et al., 2014). NAA concentration in the posterior cingulate of MCI patients has been reported to be intermediate between controls and AD patients. Applications of MRS to MCI have found broadly similar results, and additionally that choline was reduced in the hippocampus but Choline/Creatine ratio raised in the PC. Myo-inositol/creatine ratio was also raised in the hippocampus while NAA was reduced in paratrigonal WM, hippocampus and PC (Kantarci, 2013; Tumati et al., 2013; Zhang et al., 2015).

## Summary/conclusion

Early detection and treatment of dementia is conceivable in the coming generation and remains one of the most pressing current challenges for healthcare research. The contributions from MRI are potentially substantial with, for example, automated hippocampal subfield volumetrics and DTI already having significant support for clinical application and relaxometry, perfusion imaging and MRS being active areas of research that could lead to clinical applicability. Future research directions should concentrate on optimum combination of available test for early accurate cost effective dementia diagnosis.

## Author contributions

All authors contributed to the writing and editing of the Manuscript. MK and BW produced the figures.

### Conflict of interest statement

The authors declare that the research was conducted in the absence of any commercial or financial relationships that could be construed as a potential conflict of interest.
